# Echoes of conflict: the enduring mental health struggle of Gaza’s healthcare workers

**DOI:** 10.1186/s13031-024-00577-6

**Published:** 2024-03-14

**Authors:** Muna Abed Alah

**Affiliations:** Clinical Effectiveness Department, Primary Health Care Corporation, Doha, Qatar

**Keywords:** Mental health, Health personnel, Armed conflicts, Middle East, Stress disorders, Post-traumatic

## Abstract

The conflict in Gaza presents distinct difficulties that significantly impact the psychological well-being of healthcare workers (HCWs) making it imperative to understand and address their mental health needs in this specific context. This article highlights the unique challenges of the ongoing Gaza conflict and its critical impact on the mental health of HCWs. Observations in the paper revealed that HCWs in Gaza face extraordinary challenges, including the targeting of medical facilities, severe shortages of medical supplies, and the ethical dilemmas of providing care in such constrained conditions. These factors contribute to heightened stress, anxiety, and a pervasive sense of helplessness among HCWs. The paper also notes the compounded emotional burden due to the loss of colleagues and the need to navigate complex interactions with patients’ families under extreme conditions. Furthermore, the lack of basic needs like adequate nutrition and safe drinking water for HCWs themselves further compromises their ability to provide care effectively, contributing further to worsened mental health. The paper also notes the lack of sufficient media coverage and support for these workers, contributing to a sense of isolation and neglect. HCWs in Gaza find themselves in a uniquely challenging situation, one that is marked not only by the immediate stresses of the ongoing conflict but also by the deep-seated psychological scars from past wars. The circumstances in Gaza are clinically relevant as they directly affect the HCWs’ ability to provide care and maintain their well-being. These findings highlight the need for targeted mental health interventions and support tailored to the specific challenges faced by HCWs in Gaza. Addressing these issues is crucial for their well-being and ability to provide effective healthcare under such demanding and traumatic circumstances.

## Introduction

In conflict settings, the mental health of healthcare workers (HCWs) is profoundly affected by the unique and relentless challenges they face [1]. Operating in environments marked by violence, uncertainty, and resource scarcity, these individuals endure not only physical risks but also significant psychological strains [2]. The constant exposure to trauma, coupled with the pressure of providing care under extreme conditions, places an immense burden on their mental well-being, making their situation in these conflict zones both critical and often overlooked.

HCWs in conflict zones are accustomed to working under extreme pressure. However, the current situation in Gaza has pushed these limits. HCWs are working relentlessly, often for many days without a break. The sheer volume of casualties and the severity of injuries in this conflict have surpassed any previous war, setting the stage for a heightened risk of mental health issues, particularly post-traumatic stress disorder (PTSD) [3, 4]. The adverse mental health impacts of war on HCWs can persist for years, long after the immediate conflict has subsided. This enduring effect was starkly highlighted in a study previously conducted among HCWs in the Gaza Strip. The study revealed a remarkably high level of PTSD among the participants, with 89.3% still exhibiting symptoms two years after the 2014 war [5]. This paper sheds light on the psychological toll of the current war on these vital workers, highlighting the unique and severe conditions they endure.

## Unique challenges of the current Gaza conflict that contribute to worsening mental health of HCWs

The current conflict in Gaza presents a set of peculiar challenges, distinguishing it from previous conflicts in both intensity and impact. This war has unfolded in a context that brings to the forefront a range of unprecedented difficulties, particularly for HCWs who are navigating an extraordinarily complex and hazardous landscape. These challenges are not only a reflection of the immediate violence and instability but also of the deeper, systemic issues that have been exacerbated by the ongoing conflict.

### Crisis in care: hospital targeting and medical supply shortages

In the current Gaza conflict, the deliberate targeting and besieging of hospitals have emerged as alarming tactics, profoundly affecting HCWs [6]. These attacks and sieges disrupt essential healthcare services and create a climate of fear and uncertainty. Healthcare workers are forced to operate in conditions where their safety and the safety of their patients are constantly at risk. This ongoing threat not only endangers their physical well-being but also inflicts significant psychological trauma [7]. The psychological impact on them in such an environment is immense. They face the dual burden of trying to provide care in severely compromised settings while coping with the constant fear of further attacks. This situation leads to heightened levels of stress, anxiety, and a pervasive sense of helplessness. The erosion of the sanctity of medical facilities in war zones adds to the emotional toll, challenging the resilience and mental health of these frontline workers [8].

The targeting of ambulances and paramedics in Gaza is a grave concern with far-reaching implications [9]. These attacks not only endanger the lives of these frontline responders but also hinder the timely evacuation and treatment of the injured. Beyond the immediate physical risks, there is a profound emotional and psychological impact when HCWs lose their colleagues. The grief and trauma of witnessing fellow paramedics and healthcare staff being injured or killed in the line of duty are immense [10]. These losses are not just personal; they represent a weakening of every aspect of the healthcare system in times of dire need.

A critical aspect exacerbating the mental health crisis among HCWs in Gaza is the severe limitation on aid and the entry of medical supplies. The conflict has led to a near-complete blockade, severely restricting the flow of essential medical supplies and humanitarian aid into the region [11]. The scarcity of medical supplies means that HCWs are often forced to make impossible choices, rationing care for those most in need while others suffer [12]. This situation is distressing for any medical professional, as they are trained and committed to saving lives. The lack of basic medical necessities like antibiotics, surgical supplies, and even anesthetics means that treatable conditions can become fatal.

During the recent conflict in Gaza, surgeons have faced unimaginable challenges, including performing surgeries, such as wound suturing, and amputations, without anesthesia [13]. This dire situation not only reflects the extreme scarcity of medical supplies but also places an extraordinary emotional and ethical burden on the surgeons. Patients undergoing such procedures experience intense pain and suffering, a distressing reality for both the patient and the medical staff. The surgeons, trained to alleviate pain and save lives, find themselves in a paradoxical situation where they must inflict pain to provide life-saving treatment. Witnessing the pain of their patients during these procedures, knowing that this could have been mitigated under normal circumstances, is deeply troubling, often leading to feelings of helplessness.

The situation is further aggravated by the severe shortage of fuel, essential for operating hospitals [14]. Power outages and the inability to run critical medical equipment hamper healthcare delivery. In a region where electricity is already scarce, the lack of fuel for generators means that life-saving procedures cannot be performed, and essential services like refrigeration for medicines and blood supplies are compromised. This adds an additional layer of despair for HCWs, as they watch patients suffer due to factors entirely beyond their control. They are witnessing a higher rate of mortality and morbidity due to these shortages, which can lead to feelings of guilt, anxiety, and a sense of professional inadequacy. The constant battle to save lives with limited resources can lead to burnout, depression, and even secondary traumatic stress [15, 16]. The lack of adequate medical supplies not only impacts the immediate healthcare needs but also hinders the ability to provide ongoing care for chronic conditions (like chemotherapy for cancer patients or dialysis for renal failure patients) and rehabilitation for those injured in the conflict creating a cumulative effect on the mental health of HCWs, as they struggle with the realities of a healthcare system strained beyond its limits [17].

The scarcity of medical supplies and medicine in Gaza’s conflict zone extends beyond patient care, significantly impacting HCWs themselves. When these workers fall ill, they face the same shortages and limitations as their patients. This lack of access to essential medications and medical care for their own health needs adds an additional layer of vulnerability.

### Staffing strains: navigating the challenge of workforce shortages

The targeting of healthcare staff leads to a further shortage of skilled personnel. This shortage places an overwhelming burden on the remaining staff, who are already stretched due to the high demands of the conflict. The increased workload, coupled with the emotional toll of losing colleagues, significantly contributes to burnout among them [16]. They are forced to work longer hours under stressful conditions, often without adequate support or time to process their grief and trauma.

Burnout among HCWs in this context is not just about physical exhaustion; it encompasses emotional and mental fatigue as well [7]. The relentless pressure to deliver care in an environment of scarcity, danger, and personal loss can lead to feelings of despair, detachment, and a diminished sense of personal accomplishment. This state of burnout not only affects their mental health but also impacts the quality of care they can provide to patients [18].

This relentless threat and the resulting staff shortages create a vicious cycle. As more HCWs face burnout or leave the field due to the risks, the strain on the remaining staff intensifies. This situation exacerbates the mental health challenges for these workers, who are committed to saving lives under the most challenging circumstances. The constant state of high alert, grief, and overwork can lead to long-term psychological issues, including PTSD, anxiety, and depression [18].

### Under threat: the arrest of HCWs during Gaza’s conflict

The arrest of HCWs in Gaza’s conflict adds a critical challenge to an already dire situation [19, 20]. These arrests not only disrupt medical services by directly removing essential personnel but also contribute to a pervasive atmosphere of fear and uncertainty among the remaining staff. This heightened sense of insecurity can deter healthcare professionals from continuing their work in such high-risk conditions, exacerbating the existing problem of staff shortages.

The psychological impact of these arrests on HCWs is profound. The fear of being detained not only affects their mental well-being but also influences their decision-making and ability to provide care. This constant state of apprehension can lead to increased stress, anxiety, and a sense of helplessness, further contributing to the risk of burnout and other mental health issues [7]. The cumulative effect of these factors significantly hampers the overall functioning and efficacy of the healthcare system in Gaza during a time of critical need.

### Crisis of capacity: addressing hospital overcrowding amidst displacement

During the recent conflict in Gaza, an additional layer of complexity was added to the already dire situation in hospitals: the influx of forcibly displaced people seeking shelter [21]. Many individuals and families, having lost their homes or fleeing from unsafe areas, found refuge in hospitals, perceiving them as relatively safer places. This situation led to extreme overcrowding, further complicating the challenges faced by HCWs. With the presence of displaced people in hospitals, the already limited space was further constrained. HCWs found it increasingly difficult to navigate through crowded facilities, impacting their ability to work efficiently. The overcrowding also meant that finding a quiet space or a room to rest and recover became nearly impossible, adding to their physical and mental exhaustion [22].

The overcrowded conditions also compromised the quality of care that could be provided. With more people in the hospital, the risk of infection increased, and the ability to maintain a sterile environment was challenged [23]. Additionally, the presence of large numbers of non-patients created logistical issues, such as increased noise and disruptions, which could interfere with the delivery of care. These factors collectively pose a significant challenge to the mental health of HCWs, as they grapple with the dissatisfaction of not being able to provide the standard and quality of care they strive for under such constrained circumstances.

### Familial expectations: navigating the emotional demands of patients’ families

In the war-torn environment of Gaza, HCWs not only face the challenges of limited resources and personal safety concerns but also encounter significant pressure from the families of the injured. These families, often in a state of distress and anxiety, naturally expect that their loved ones will receive full and comprehensive care. However, given the scarcity of medical supplies and the overwhelming number of casualties, providing such care is frequently beyond the capabilities of the healthcare system. Healthcare workers find themselves navigating a complex maze of emotional and ethical challenges as they must often communicate with families about the limitations in care due to the lack of resources, and they need to break bad news to families which can be a profoundly distressing task [12, 24].This responsibility, often a recurring part of their job in conflict zones, involves not only the delivery of the news itself but also managing the emotional reactions that follow. Dealing with families who are grieving, fearful, and sometimes frustrated or angry due to the circumstances further exacerbates the issue.

### Balancing professional commitments and personal worries

HCWs in Gaza are not only worried about their patients but also live in constant fear for their families and homes, given the extensive destruction witnessed in this war. While HCWs tend to the injured and sick, their minds are often with their families, who are vulnerable to the dangers of conflict [25]. This constant worry about the safety of their loved ones is a heavy burden that they carry even as they perform their critical duties. The nature of their work often requires healthcare workers to be separated from their families for extended periods, especially during intense phases of the conflict. This separation, coupled with the uncertainty of not knowing if their families are safe, can be emotionally taxing. The lack of reliable communication channels in a war zone exacerbates this anxiety, as getting updates about their families’ safety can be challenging. Despite these overwhelming personal concerns, they strive to maintain a level of professionalism and focus on their patients. However, the emotional toll of worrying about their families cannot be understated.

### Compromised well-being: the impact of basic needs shortage on HCWs

The absence of adequate nutrition and safe drinking water leads to physical weakness, making it increasingly difficult for them to perform their duties effectively. Physical strength and well-being are crucial not only for the demanding physical tasks they must undertake but also for maintaining clear thinking and optimal mental health [26].

In such an environment, where even the most basic needs are unmet, the physical and mental health of HCWs is severely compromised. This creates a vicious cycle where their ability to provide care is hindered not just by external factors like the lack of supplies and overcrowding, but also by their own deteriorating health and well-being. The need for comprehensive support that addresses both their physical and mental health needs in these conflict zones is therefore of paramount importance.

### Neglected voices: the overlooked struggles of HCWs

During the ongoing conflict in Gaza, there is a noticeable lack of emphasis in the media on the needs and situation of HCWsthemselves. This oversight contributes to a feeling among these workers of being overlooked and undervalued, despite the critical role they play and the immense challenges they face leading to a detrimental effect on their mental health. Moreover, they have expressed feelings of abandonment by humanitarian organizations and reported a sense of isolation and neglect, and felt that the support and resources typically provided by these organizations are insufficient or absent in their time of greatest need [27]. HCWs in conflict zones like Gaza are not just professionals performing their duties; they are individuals facing extraordinary circumstances, often at great personal risk and emotional cost. The absence of adequate media coverage on their situation means that the recognition they deserve and the support they critically need are not sufficiently highlighted. This lack of awareness can impact the mobilization of international support and resources that are essential for their well-being and effectiveness.

### A prolonged crisis: the cumulative mental health challenges in Gaza

The situation in Gaza presents a unique and particularly challenging scenario, shaped by the cumulative impact of multiple wars and a prolonged siege spanning over 17 years [28]. This prolonged exposure to conflict and restrictions has created a deeply challenging environment for the population, including HCWs, who are already struggeling with significant mental health challenges. Several studies have indicated high prevalence rates of PTSD and other mental health issues among these workers [5, 29]. The constant state of alert, the recurring exposure to trauma, and the chronic stress of living in a conflict zone have contributed to a widespread mental health crisis.

## Conclusion

In conclusion, HCWs in Gaza find themselves in a uniquely challenging situation, one that is marked not only by the immediate stresses of the ongoing conflict but also by the deep-seated psychological scars from past wars. Their enduring struggle is a result of prolonged exposure to traumatic events, compounded by the current protracted crisis and siege. This complex backdrop necessitates specialized attention and support for their mental health needs, considering the cumulative impact of their repeated exposure to trauma.

The effective functioning of Gaza’s healthcare system hinges on addressing these critical needs. It is essential to recognize that the well-being of HCWs is intrinsically linked to their ability to manage the medical needs of their patients, despite the scarcity of resources and the emotional toll of dealing with distressed families and personal fears for their own families’ safety.

Moreover, the role of the media in this context cannot be overstated. By bringing more attention to the experiences and challenges faced by them in conflict zones, the media can play a pivotal role in shaping public perception and driving humanitarian responses. This increased awareness is crucial in ensuring that these vital workers receive the recognition and support they so desperately need and deserve.

Ultimately, the mental health strain on HCWs in Gaza is a multifaceted issue that requires a comprehensive and empathetic approach, one that acknowledges and addresses the complexities of their situation in a conflict-ridden environment.

## Data Availability

No datasets were generated or analysed during the current study.
